# Supplementary material for: Percutaneous Chevron/Akin (PECA) versus open scarf/Akin (SA) osteotomy treatment for hallux valgus: A systematic review and meta-analysis
Source: PLoS One. 2021 Feb 17;16(2):e0242496. doi: 10.1371/journal.pone.0242496 (PMC7888602; doi:10.1371/journal.pone.0242496)
Supplement: Attachment — Submitted filename: Reviewer_Table.docx [file pone.0242496.s002.docx]

Percutaneous Chevron/Akin versus open scarf/Akin osteotomy treatment for hallux valgus: a systematic review and meta-analysis

Response to Reviewers

A detailed response to the recommendations of the reviewers is provided here, including the specific revisions made to the original manuscript. A table format has been used for clarity.

**Reviewer 1**

| **COMMENT** | **RESPONSE** | **TEXT CHANGES** |
| --- | --- | --- |
| **General** | | |
| Thank you for asking me to review this paper dealing with a comparison between percutaneous Chevron/Akin and traditional Scarf/Akin. The topic is definitely interesting. I think the paper will be suitable for publication after a revision by authors. The most important point will be to remodulate the conclusion, since I don’t believe that data allow to say the early postoperative pain is better for PECA. It can be said that the outcomes are comparable after 6 months, but that no analysis is possible about the early recovery based on published data. | Thank you for this comment. We agree that the data is not robust enough for this conclusion. We changed the conclusion of the study as suggested by the reviewer. Thank you very much. |  |
| **Abstract** | | |
| I would recommend to include more data in the methods section. In results, how many patients were considered in the 3 studies in total? | Perfect. We added in abstract and results the total number of feet that underwent surgical treatment and the results found in the meta-analysis, valuing the data included in the three studies. The conclusion was modified according to the reviewer's suggestion. | Line 14 and Line 122-123 |
| **Introduction** | | |
| Line 59 Better to say The reviews published so far… | The sentence really got confused. We changed it as suggested. Thank you. | Line 37 |
| **Methods** | | |
| Line 85 I think here you may want to say ‘…treatment by the SA and PECA technique » | We agree with the comment. We changed the "or" to "and". It was much better that way. Thank you. | Line 63 |
| **Results** | | |
| Line 170 Is this Portuguese? | Translated. This was a mistake by the translator. We apologize and appreciate the comment. | Line 146 |
| Line 171 Typo | Adjusted. | Line 147 |
| Line 179-180 This is not clear | The item was to be clear about subsequent analyzes. But it was loose in the text and confused. Thanks for the comment. We decided to remove. |  |
| Line 188 -0.53 what? and in favour of what technique? | The grouped analysis corresponds to the difference in the averages. We standardize the calculations to be PECA minus SA, in the text is for example "(HVA PECA - HVA SA)". If the difference in the average is negative as in the case -0.53, it means that the mean of SA is greater than PECA. This comparison in means is standardized for all forest plots in the present study, as in the other values found. |  |
| Line 193 As above. Please, be clearer when reporting differences |  |  |
| Line 198 As above |  |  |
| Line 206 As above |  |  |
| Line 212 As above |  |  |
| **Discussion** | | |
| Line 233 0.80 what? Please revise the whole paper indicating the Units for values | Thank you for the question. In this case 0.80 and 0.53 are the difference in the means of the HVA and IMA angles. “...final HVA and IMA, with means derived from ...” We added the word "degrees" in the sequence of values for easy reading. | Lines: 161, 164, 169, 174, 178 |
| Line 237-243 I think this paragraph should be remodulated. Was the difference in AOFAS statistically significant? I think you cannot draw any conclusion on the immediate postoperative pain since only one or two studies assessed it. | Very good comment. We cannot affirm and conclude with absolute certainty the results, as we have points of possible bias in the study. We included in the last paragraph of the discussion: "These factors can influence the risk of bias and lead to erroneous conclusions." The result of the meta-analysis we interpret as a pooled analysis and heterogeneity (i^2^), and not on statistical significance. The difference in the means is a statistical synthesis that can present distortions that are demonstrated in the weak points of the study. |  |
| Line 247 Pain score at VAS cannot be judged only on one or two studies | In fact, the low number of articles included in the meta-analysis can increase the risk of bias and restrict its generalization. We add in this paragraph that the result was obtained from only two studies, with this evident limitation. We appreciate the suggestion. | Line 223 - 224 |
| **Conclusion** | | |
| I think that, based on your numbers, you cannot infer that PECA led to less postoperative pain. You may say that the clinical outcome is comparable at 6 and 12 months, and that early recovery cannot be assessed based on current literature. | The conclusion was modified according to the reviewer's suggestion, both in the abstract and in the conclusion. | Line 253 – 255 and Line 19 - 21 |

**Reviewer 2**

| **COMMENT** | **RESPONSE** | **TEXT CHANGES** |
| --- | --- | --- |
| This study addresses a common problem in foot surgery. A well-tried technique is compared with a new percutaneous technique in the context of a meta-analysis. This analysis was carried out technically flawlessly. The only problem I see is that only 3 studies could be included. However, this fact was openly presented and discussed by the authors in the discussion. | We really appreciate your time for this review. We hope that after the modifications suggested by the reviewers, the article will be suitable for publication. Thank you very much. |  |
| Please delete „were excluded“ in line 87 | Thank you for the comment. The word "excluded" was really inappropriate. We changed it to "removed". Thank you. | Line 65 |
